# Sweet syndrome associated with moderate leukocyte adhesion deficiency type I: a case report and review of the literature

**DOI:** 10.3389/fimmu.2024.1425289

**Published:** 2024-07-16

**Authors:** Yoshine Saito, Anupama Kewalramani, Xiao P. Peng, Aimee Magnarelli, Howard M. Lederman

**Affiliations:** ^1^ University of Maryland School of Medicine, Baltimore, MD, United States; ^2^ Department of Pediatrics, Division of Pulmonology/Allergy, University of Maryland School of Medicine, Baltimore, MD, United States; ^3^ Department of Genetic Medicine, Johns Hopkins University School of Medicine, Baltimore, MD, United States; ^4^ Eudowood Division of Pediatric Allergy, Immunology and Rheumatology, Johns Hopkins University School of Medicine, Baltimore, MD, United States

**Keywords:** inborn error of immunity (IEI), sweet syndrome, neutrophilic dermatosis, leukocyte adhesion deficiency type 1, whole exome sequencing

## Abstract

Sweet syndrome is an acute febrile neutrophilic dermatosis characterized by the infiltration of neutrophils into the skin. It may occur idiopathically or be linked to malignancies, inflammatory or autoimmune diseases. Leukocyte adhesion deficiency type I (LAD-I) is an inborn error immunity wherein leukocytes lack adhesion molecules necessary for migration to infection sites due to mutations in the CD18 gene encoding β2 integrins. We present a case of a 16-month-old female initially diagnosed and treated for Sweet syndrome based on histopathological findings with recurrent flare episodes. Subsequent workup revealed LAD-I, making this case the first documented association between Sweet syndrome and LAD-I. Moreover, we reviewed the pertinent literatures detailing the concurrence of neutrophilic dermatosis and immunodeficiency disorders. This case underscores the significance of comprehensive evaluation for Sweet syndrome patients who are refractory to conventional treatments.

## Introduction

Sweet syndrome is a neutrophilic dermatosis associated with an abrupt onset of tender plaques and nodules that can be accompanied by fever and leukocytosis. Although rare in children, Sweet syndrome can accompany viral infections, autoimmune processes, and inborn errors of immunity (IEI). While Sweet syndrome has been described in patients with IEI, this is the first report of the neutrophilic skin eruption occurring in association with leukocyte adhesion deficiency (LAD). LAD type I, the most common adhesion deficiency, occurs in 1 in 1,000,000 individuals. This condition arises from mutations in *ITGB2*, the gene encoding the β subunit (CD18) of the β2 integrins, leading to recurrent, life-threatening bacterial and fungal infections. Although these patients have neutrophilia, the cells are unable to migrate to tissues in which infection and inflammation are present. As such, biopsies of inflammatory or infectious sites lack neutrophils. Herein, we describe a patient initially diagnosed and treated as Sweet syndrome; however, with recurrent and uncontrolled skin findings on treatment, further evaluation led to a diagnosis of LAD type I.

## Case presentation

**Figure 1:**
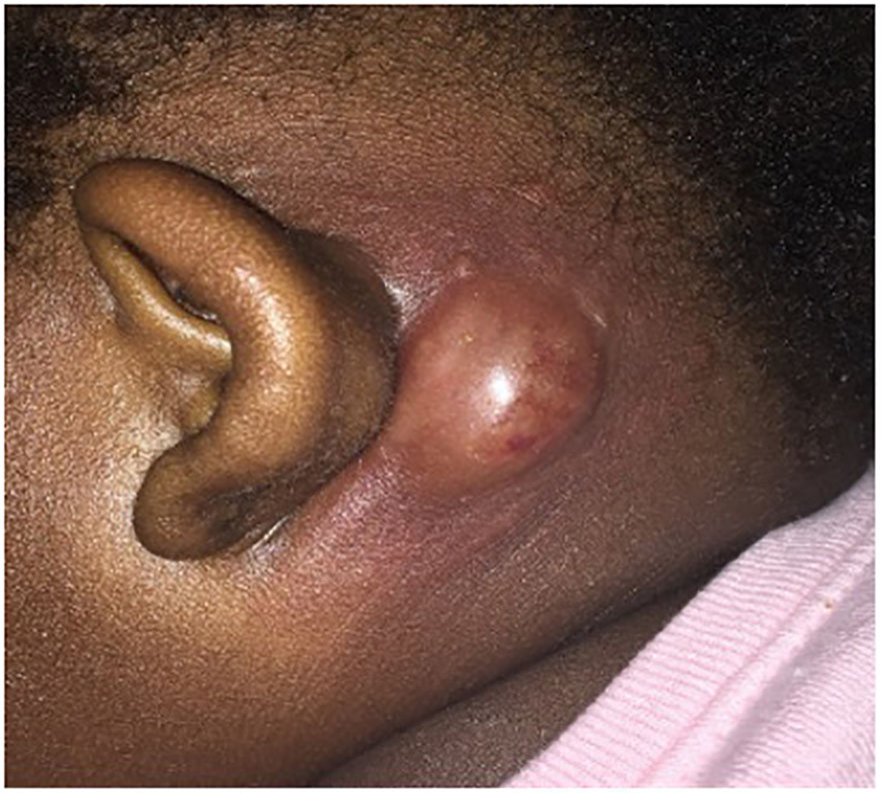
Left post-auricular mass. Left ear proptosis with an erythematous, tender post-auricular fluctuant mass.

A 16-month-old African American female presented to our hospital with 4 days of left ear proptosis with an erythematous, tender post-auricular fluctuant mass and intermittent fever that was not improving despite 4 days of outpatient clindamycin. The patient was born full-term by cesarean section with no post-partum issues. No delayed umbilical cord separation was reported. Early infancy was unremarkable without developmental concerns or dietary restrictions. The patient has three half-siblings and one full sibling, all of whom are healthy. The patient’s past medical history includes atopic dermatitis and a 2-month history of tinea capitis on griseofulvin. About 3 weeks before the onset of the post-auricular mass, the patient was hospitalized twice for respiratory distress ascribed to croup, requiring multiple treatments of racemic epinephrine. Physical examination on presentation revealed an erythematous, fluctuant lump behind the left ear that was tender and warm with cervical lymphadenopathy (**Figure 1**). The induration and erythema did not extend to the pinna, and the tympanic membrane and ear canal were normal. Cardiopulmonary and abdominal exams were unremarkable. Laboratory tests were significant for an elevated white blood cell count (WBC) of 19,000/µL (normal: 10.0 - 30.0 K/µL), 45.9% segmented neutrophils (normal: 35.0 - 70.0%), 7.0% bands (normal: 0.0 - 5.0%), and absolute neutrophil count (ANC) of 10170/µL (normal: 3.50 - 22.0 K/µL). C-reactive protein (CRP) was 9.3 mg/dL (normal: ≤ 1.0 mg/dL). Computed tomography (CT) scan of the temporal bone and ultrasound were performed, which revealed an abscess without periosteal reaction or osseus destruction. She was treated with intravenous (IV) vancomycin.

**Figure 2:**
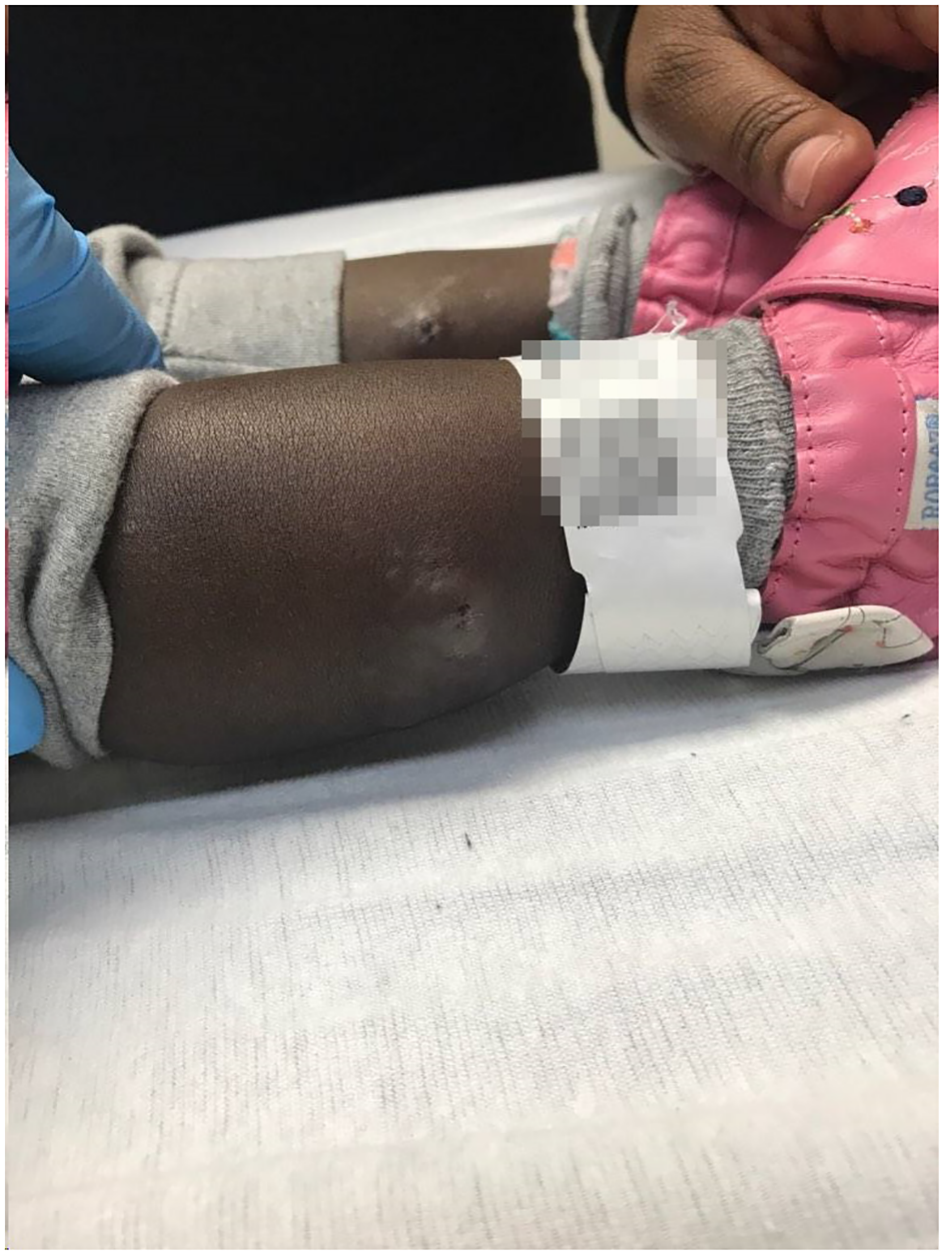
Sweet syndrome skin lesions. Right and left posterior calf subcutaneous nodules with a central necrotic opening.

**Figure 3:**
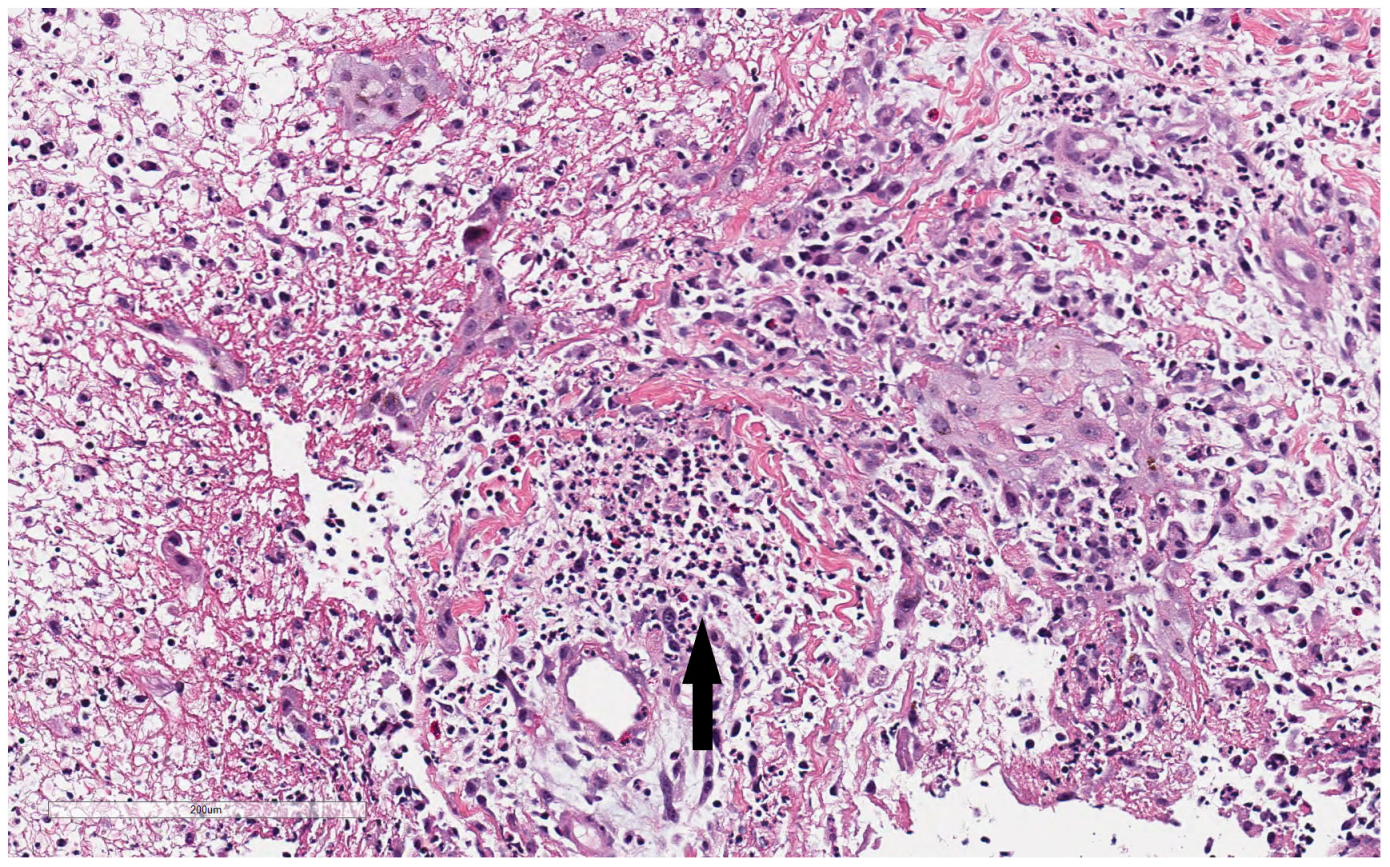
Histopathologic findings from patient’s right calf biopsy. Biopsy from the right calf lesion revealed dense dermal neutrophilic infiltration (black arrow), increased eosinophils, and perivascular chronic inflammation without evidence of infection.

During the hospitalization, the patient developed numerous new lesions on the right posterior neck, mid-back, right and left posterior calves (**Figure 2**), and left gluteal fold despite antibiotic therapy. These lesions appeared as firm violaceous subcutaneous nodules, some with central necrosis. Culture from an incision and drainage (I&D) of the left post-auricular lesion demonstrated abundant polymorphonuclear leukocytes with no organism. Due to the poor response to antibiotics, the patient underwent an immunodeficiency work-up; however, results were grossly normal except for elevated IgE of 2233 KU/L (normal: ≤ 97 KU/L). **Table 1** summarizes the laboratory test results. To assess the newly developed skin lesions, she underwent a right calf skin punch biopsy. The pathology from a specimen containing only epidermis and dermis revealed a dense dermal neutrophilic infiltration, increased eosinophils, and perivascular chronic inflammation without evidence of infection, suggestive of acute febrile neutrophilic dermatosis, also known as Sweet syndrome (**Figure 3**). Three weeks after initial admission, the patient was seen by Dermatology for concern for poor wound healing of the I&D site for which they recommended applying silver-carboxymethylcellulose dressings to the area.

**Table 1:** Laboratory test results of the patient with reference ranges.

Lab Finding	Value	Reference ranges & units
WBC	22.8	6.0 – 17.5 K/mL
Hemoglobin	10.1	10.5 – 13.5 g/dL
Hematocrit	31.4	33.0 – 39.0%
Platelets	308	153 – 367 K/mL
Neutrophils Absolute	11.83	1.70 – 7.30 K/mL
Neutrophils %	50	26 – 50%
Bands %	2	0 –2%
Sodium	136	136 – 145 mmol/L
Potassium	–	4.1 – 5.3 mmol/L
Chloride	99	98 – 107 mmol/L
CO2	26	21 – 30 mmol/L
BUN	2	7 – 17 mg/dL
Creatinine	0.15	0.22 – 0.63 mg/dL
Glucose	101	70 – 99 mg/dL
AST	–	14 – 36 units/L
ALT	16	0 – 34 units/L
Bilirubin total	0.7	0.3 – 1.2 mg/dL
Alkaline phosphate	172	145 – 320 units/L
CRP	4.8	≤ 1.0 mg/dL
CD19 absolute	1568	160 – 3700 cells/mL
CD19%	22	8 – 45%
CD3 absolute	4711	700 – 8800 cells/mL
CD3%	65	36 – 92%
CD4 absolute	3670	400 – 7200 cells/mL
CD4%	50	16 – 91%
CD4:8 ratio	4.55	0.9 – 3.7 Ratio
CD8 absolute	795	200 – 2800 cells/mL
CD8%	11	7 – 40%
NK (CD56) %	13	1 – 96%
NK absolute	976	55 – 4400 cells/mL
IgG	750	468 – 1196 mg/dL
IgA	24	21 – 117 mg/dL
IgM	90	47 – 200 mg/dL
IgE	2233	≤ 97 KU/L
Complement total (CH50)	99	60 – 144 CAE Units
NADPH oxidase activity	Normal	Normal
Diphtheria Tox IgG Ab	0.5	≥ 0.01 IU/mL
Hemophilus influenzae b Ab	106.6	≥ 0.15 mg/mL
Pneumococcal Type 1 Ab	8.29	≥ 1.3 mg/mL
Pneumococcal Type 3 Ab	9.06	≥ 1.3 mg/mL
Pneumococcal Type 4 Ab	9.25	≥ 1.3 mg/mL
Pneumococcal Type 5 Ab	2.51	≥ 1.3 mg/mL
Pneumococcal Type 6B Ab	8.23	≥ 1.3 mg/mL
Pneumococcal Type 7F Ab	1.26	≥ 1.3 mg/mL
Pneumococcal Type 8 Ab	0.23	≥ 1.3 mg/mL
Pneumococcal Type 9N Ab	1.76	≥ 1.3 mg/mL
Pneumococcal Type 9V Ab	10.61	≥ 1.3 mg/mL
Pneumococcal Type 12F Ab	0.04	≥ 1.3 mg/mL
Pneumococcal Type 14 Ab	2.40	≥ 1.3 mg/mL
Pneumococcal Type 18 Ab	4.70	≥ 1.3 mg/mL
Pneumococcal Type 19F Ab	17.54	≥ 1.3 mg/mL
Pneumococcal Type 23 Ab	1.59	≥ 1.3 mg/mL
Tetanus Antitoxoid IgG Ab	17.3	≥ 0.01 IU/mL

WBC, white blood cell; BUN, blood urea nitrogen; AST, aspartate transaminase; ALT, alanine transminase; CRP, C-reactive protein; NK, natural killer; NADPH, nicotinamide adenine dinucleotide phosphate; Ab, antibody.

One year later, the patient presented with 1 day of worsening swollen, erythematous, tender left thumb paronychia that progressed to full left-hand swelling. Until this encounter, the patient was healthy without recurrent or serious infections. Her skin exam was also notable for keloids on bilateral arms at previous IV sites, three small non-tender lesions with minimal yellow drainage on the central abdomen, and excessive scarring at sites of previous lesions across the body. No other abnormalities were noted on physical exam and abdominal ultrasound revealed no hepatosplenomegaly. She was subsequently hospitalized, where laboratory tests revealed a WBC count of 39,200/µL with an ANC of 31,790/µL, and CRP of 19.4 mg/dL. A bedside I&D was performed, and the patient was treated with IV clindamycin for methicillin resistant *Staphylococcus aureus* (MRSA) cellulitis in the setting of paronychia. Despite antibiotic therapy and I&D, she continued to be febrile throughout hospitalization and developed a new abscess on the dorsal aspect of the left hand which required drainage. She also developed erythematous nodular skin lesions on her scalp, abdomen and bilateral upper and lower extremities, like her previous hospitalization. Due to recurrent fever and significant leukocytosis, Hematology/Oncology was consulted to assess for the possibility of malignancy. A bone marrow biopsy was not done because the peripheral smear was only notable for abundant neutrophils with reactive monocytes and no evidence of peripheral blasts. The patient was started on oral prednisolone 0.5 mg/kg twice a day for treatment of a Sweet syndrome exacerbation in the setting of a MRSA skin infection. After 5 days of steroid treatment, symptoms were effectively controlled. She was afebrile and no new skin lesions developed. She was subsequently discharged from the hospital on a prednisolone taper.

The patient followed up with Dermatology as an outpatient. She continued to have diffuse firm, tender, fluid-filled nodules. These lesions often had surrounding induration and central fluctuance and recurred approximately every three months, typically triggered by an upper respiratory infection or ear infection. One such flare resulted in another hospitalization that required IV antibiotics and IV steroids to control her skin lesions. As these Sweet syndrome flares typically responded to systemic steroids, the patient was advised to use triamcinolone 0.1% ointment for mild flares and prednisolone taper for severe flares at home. The patient was also evaluated by a different dermatologist for a second opinion who started her on oral colchicine as a steroid-sparing agent and topical clobetasol 0.05% for long-term management. However, the patient subsequently presented with new, tender nodules on the face, bilateral upper and lower extremities that looked distinct from previous Sweet syndrome skin lesions, warranting a repeat biopsy and prednisolone taper. The pathology of this biopsy, which did not include subcutaneous tissue examination, revealed a neutrophilic and reactive granulomatous infiltrate with a more pronounced histiocytic component. Combining the patient’s history of refractory diffuse skin lesions triggered by antecedent illness and histopathological results, the patient was referred to Genetics for further evaluation of autoinflammatory dermatoses.

The patient’s comprehensive family history was largely unrevealing; the patient’s mother had no history of recurrent stillbirths, miscarriages, or congenital anomalies. No known hereditary disorders were found in the first-degree relatives, and no consanguinity. The patient and her parents underwent a whole exome sequence (WES) and chromosomal microarray studies. The WES result demonstrated a compound heterozygosity for two variants in the *ITGB2* gene (c.817G>A; p.Gly273Arg and c.314T>C; p.Leu105Pro) consistent with the molecular diagnosis of autosomal recessive leukocyte adhesion deficiency type I (LAD-I). The c.817G>A variant, in the exonic region, causes a p.G273R amino acid change and results in a missense mutation. According to gnomAD v4.0.1, there are 101 heterozygotes and 0 homozygotes among 1,613,960 alleles. In All of Us, 17 heterozygotes and 0 homozygotes have been reported among 490,758 alleles. The Rare Exome Variant Ensemble Learner (REVEL) predicts this variant to be strongly pathogenic (score 0.955), and the Combined Annotation Dependent Depletion (CADD) score is 29.5. The c.314T>C variant, also in the exonic region, leads to a p.L105P amino acid change resulting in another missense mutation. In gnomAD v4.0.1, there are 73 heterozygotes and 0 homozygotes among 1,613,988 alleles. In All of Us, 87 heterozygotes and 0 homozygotes have been reported among 490,758 alleles. The REVEL predicts pathogenic supporting (score 0.789), and the CADD score is 24.7.The patient’s younger sister, who shares the same mother and father as the patient, did not demonstrate either of the two *ITGB2* gene mutations in WES.

The patient was referred to Pediatric Immunology, who felt that even without CD18 flow cytometry, a diagnosis of LAD-I was established based on her genetic work-up and presentation. A hematopoietic stem cell transplant (HSCT) from either parent who are haploidentical donors was recommended as the best long-term treatment option. However, the patient has since done well without infections after implementing weekly bleach baths to decrease the bacterial burden on the skin. Most recently, the patient’s physical exam was significant for gingival inflammation without gingival recession.

## Discussion

A thorough search of the relevant literature reveals that this is the first reported case of LAD-I accompanied by a diagnosis of Sweet syndrome (SS). The patient demonstrated “a dense dermal neutrophilic infiltration” on a skin biopsy. As such, the patient was initially diagnosed and treated as SS. However, typical treatments did not lead to a durable response which led to a referral to Genetics and a subsequent diagnosis of LAD-I.

LAD comprises a group of rare autosomal recessive conditions that occur due to a defect in leukocyte adhesion. LAD-I, the most common subtype of LAD, occurs in 1 in 1,000,000 births. It is associated with mutations in *ITGB2*, the gene encoding the β subunit (CD18) of the β2 integrins. These mutations lead to decreased expression or activity of β2 integrin in polymorphonuclear leukocytes which affects their adhesion to other cells and extracellular matrix proteins. Consequently, leukocytes are unable to firmly adhere to the endothelium and migrate to sites of infection and inflammation. LAD-I patients are unable to effectively prevent bacterial and fungal infections as their neutrophils amass in the blood stream but fail to reach sites of infections in tissues (1, 2).

The severity of the presentation and outcomes are related to the degree of CD18 deficiency on the surface of leukocytes (3). Severe LAD-I, classified as having < 2% of CD18-expressing neutrophils, is evident early in infancy with umbilical cord complications, such as delayed cord separation and/or omphalitis. Infant mortality can be high without early recognition as patients develop recurrent life-threatening bacterial or fungal infections with impaired pus formation. Patients will commonly exhibit upper and lower respiratory tract infections such as otitis media and pneumonia (4, 5). Skin findings including perianal and abdominal wall abscesses, fasciitis, dactylitis, cellulitis and impaired wound healing are also typical presentations (4, 6, 7). Moreover, as patients age, they may experience inflammatory complications such as severe periodontitis with bone resorption and premature loss of primary and permanent teeth (8, 9). Despite the paucity of neutrophils in affected tissues, patients usually have mild to moderate neutrophilia (10). The circulating neutrophil count during infection can be higher than 30,000/mL and can even exceed 100,000/mL (4, 11). In LAD-I patients, the paucity of neutrophils in tissues (e.g., oral mucosa, skin and gastrointestinal tract) results in excessive interleukin-23 (IL-23) expression by macrophages leading to increased interleukin-17 (IL-17) and granulocyte-colony stimulating factor (G-CSF) levels. G-CSF overexpression induces excessive granulopoiesis and blood neutrophilia. IL-17 contributes to a potent inflammatory response associated with bone loss such as seen in periodontal disease and bacterial overgrowth (8, 9).

Patients with moderate LAD-I with 2-30% CD18-expressing neutrophils will also have elevated circulating neutrophil counts but frequently survive childhood. In Almarza-Novoa’s review of published cases of LAD-I, survival was highest in those patients with more than 4% CD18 expression by age 2 years old (at least 95%), whereas only 39% survived to the age of 2 years with severe LAD-I (4). Patients with moderate LAD-I are less likely to have delayed cord separation and omphalitis which may lead to a delay in diagnosis. Although these patients can survive into adulthood, some may develop overwhelming infections later in life. The average lifespan of patients with the less severe form of LAD-I is approximately 40 years old and as such can experience frequent skin infections and disabling, recurrent skin ulcerations, inflammatory periodontal disease with bone loss and/or inflammatory bowel disease (9, 12).

Patients with the severe form of LAD-I have a poor prognosis without early hematopoietic stem cell transplantation (HSCT) (4). Moderate LAD-I patients should be aggressively treated with antibiotics and antifungals when infected and prophylaxis should be discussed to prevent recurrent skin ulcerations with scarring and overwhelming infection (10, 13). Considering the progressive course of the disease, even in its moderate form, treatment with HSCT should be considered for these patients as well (6). However, newer treatments have become available. A 19-year-old with LAD-I accompanied by significant periodontal disease and a sacral wound was successfully treated with ustekinumab, an antibody that binds the p40 subunit of IL-23 and interleukin-12 (IL-12) (14). Treatment such as this may allow for longevity in LAD-I patients.

Sweet syndrome, a clinicohistopathologic diagnosis, is a neutrophilic dermatosis first described by Dr. Robert Douglas Sweet in 1964 (15). It is frequently seen in adult females, but children and adolescents make up 5 to 8% (16) of cases. It is characterized by an abrupt onset of tender plaques and nodules frequently seen with fever and neutrophilia (17, 18). Pediatric patients may also present with vesicles, pustules, bullae, oral ulcers and pathergy (19). The most common areas of skin affected are the extremities, head, and face. Biopsies of affected areas demonstrate a dense dermal infiltrate predominantly composed of mature neutrophils and papillary edema. Likewise, infectious stains and cultures are usually negative (15). SS can be idiopathic (classic), malignancy-associated or drug-induced. Although regarded as idiopathic, up to 42% of pediatric SS cases occur following an acute infection, typically otitis media and viral upper respiratory or gastrointestinal infections. Approximately one-third of cases are associated with chronic inflammatory conditions including autoimmune diseases such as inflammatory bowel disease and systemic lupus erythematosus. Moreover, there have been several reports of SS occurring in children with IEIs, either before or after the diagnosis of the IEI was made. Sweet syndrome has been described in association with common variable immunodeficiency (CVID) and chronic granulomatous disease (CGD), but not LAD-I (19–25). Pyoderma gangrenosum (PG), another non-infectious neutrophilic dermatosis, has also been described as a comorbidity of IEI, including LAD-I (3, 6, 11, 12, 26). All neutrophilic dermatoses occurring in pediatric patients should incite clinicians to examine patients for evidence of involvement of other organs and other general disorders (27).

Although CD18 flow cytometry was not performed in our patient, based on her clinical course and concomitant diagnosis of SS, she most likely has a moderate form of LAD-I. The patient’s lack of delayed cord separation during infancy, recurrent skin blistering and ulceration without deadly infections, recent gingival changes noted in the physical exam are all consistent with the moderate presentation of LAD-I. We would not expect patients with severe forms of LAD-I to be capable of demonstrating a “dense dermal neutrophilic infiltration” on a skin biopsy. Her initial admission was accompanied by a mild elevation in her neutrophil count, but she subsequently demonstrated an elevated neutrophil count surpassing 30,000/mL with her second admission. She was found to have a MRSA infection which may have explained the initial elevation, but the persistent neutrophilia led to the continued consideration of SS. She did clinically improve with a steroid taper; yet continued to have flares that were not responsive to steroids.

This case emphasizes the value of genetic testing for patients with unusual findings, particularly when they occur early in life, in the context of a family history or consanguinity. The diagnosis of LAD-I was made in this case by WES that revealed compound heterozygosity for two variants in the *ITGB2* gene. The lack of homozygosity for the mutations in public databases corroborates the relevance of the variants.

The c.817G>A variant has been reported in homozygosity in several unrelated patients with LAD-I but has not been observed in homozygosity in controls (5, 28). Functional studies have demonstrated a damaging effect through a lack of expression of LFA-1 on the surface of cells expressing this variant (28, 29). *In silico* analyses further support a deleterious effect on protein structure and function, including a potential effect on splicing. This variation is not found at significant frequency in large population cohorts. It is classified as pathogenic/likely pathogenic by three independent submitters. This mutation results in a nonconservative physiochemical change in a highly conserved residue.

The c.314T>C variant is classified as likely pathogenic based on a previous report of homozygosity in an LAD-I patient presenting with a similar clinical phenotype as our patient, but it has not been seen in homozygosity in controls (13). Functional studies suggest that this predicted missense mutation impairs cell surface expression of β2 integrins (30). *In silico* analyses support a deleterious effect on protein structure and function, including a potential impact on splicing. This variation is found in large population cohorts with the highest subpopulation minor allele frequency (0.1%). Notably, heterozygosity for this variant is reported for 42 individuals in gnomAD genomes and 29 individuals in gnomAD exomes. In each case, all but one of the individuals is of African/African American ancestry, suggesting the possibility of this being a founder mutation. The most recent classification of the variant in ClinVar is likely pathogenic and uncertain significance. This variant is predicted to result in a conservative physiologic change but conferring more steric rigidity in a highly conserved residue.

The patient has been doing well without significant flares or hospitalization with the use of bleach baths, which her parents think have significantly improved her quality of life. In the meantime, the patient’s parents are weighing the risks and benefits of HSCT. In moderate cases of LAD-I like our patient, patients should be monitored for further signs and symptoms, including gastrointestinal, periodontal and skin problems. In case overwhelming skin lesions return, a more aggressive antibiotic regimen and immunosuppressives, including oral steroids or IL-12/23 inhibitors, will be considered as temporizing measures. However, HSCT remains the best intervention for increasing longevity.

This case emphasizes the rarity of Sweet syndrome in children and if present should prompt the practitioner to perform a comprehensive examination for systemic disorders, including inborn errors of immunity.

## Data availability statement

The original contributions presented in the study are included in the article/supplementary material. Further inquiries can be directed to the corresponding author.

## Ethics statement

Written informed consent was obtained from the minor(s)’ legal guardian/next of kin for the publication of any potentially identifiable images or data included in this article. Written informed consent was obtained from the participant/patient(s) for the publication of this case report.

## Author contributions

YS: Conceptualization, Writing – original draft, Writing – review & editing. AK: Conceptualization, Writing – original draft, Writing – review & editing, Supervision. XP: Writing – review & editing, Data curation. AM: Writing – review & editing, Writing – original draft. HL: Supervision, Writing – review & editing.

## References

[1] Erdem S Haskologlu S Haliloglu Y Celikencir H Arik E Keskin O. Defective Treg generation and increased type 3 immune response in leukocyte adhesion deficiency 1. Clin Immunol. (2023) 253:109691. doi: 10.1016/j.clim.2023.109691 37433423

[2] Roos D van Leeuwen K Madkaikar M Kambli PM Gupta M Matthews V. Hematologically important mutations: leukocyte adhesion deficiency (second update). Blood Cells Mol Dis. (2023) 99:102726. doi: 10.1016/j.bcmd.2023.102726 36696755

[3] Opalinska A Burdacki A Kwasniak K. Pyoderma gangrenosum with an underlying leukocyte adhesion deficiency type 1 (LAD-1) and pregnancy in the shade of COVID-19 epidemic: a patient and physician experience. Dermatol Ther (Heidelb). (2021) 11:643–53. doi: 10.1007/s13555-021-00507-x PMC7939102 33686591

[4] Almarza-Novoa E Kasbekar S Thrasher AJ Kohn DB Sevilla J Nguyen T. Leukocyte adhesion deficiency-I: a comprehensive review of all published cases. J Allergy Clin Immunol Pract. (2018) 6:1418–20. doi: 10.1016/j.jaip.2017.12.008 29371071

[5] Madkaikar M Italia K Gupta M Chavan S Mishra A Rao M. Molecular characterization of leukocyte adhesion deficiency-I in Indian patients: identification of 9 novel mutations. Blood Cells Mol Dis. (2015) 54:217–23. doi: 10.1111/pai.13990 25703682

[6] Fazlollahi MR Hamidieh AA Moradi L Shokouhi Shoormati R Sabetkis N Esmaeili B. Clinical and immunological characteristics of 69 leukocyte adhesion deficiency-I patients. Pediatr Allergy Immunol. (2023) 34:e13990. doi: 10.1111/pai.13990 37492921

[7] Kambli PM Bargir UA Yadav RM Gupta MR Dalvi AD Hule G. Clinical and genetic spectrum of a large cohort of patients with leukocyte adhesion deficiency type 1 and 3: a multicenter study from India. Front Immunol. (2020) 11:612703. doi: 10.3389/fimmu.2020.612703 33391282 PMC7772426

[8] Hajishengallis G Moutsopoulos NM. Etiology of leukocyte adhesion deficiency-associated periodontitis revisited: not a raging infection but a raging inflammatory response. Expert Rev Clin Immunol. (2014) 10:973–5. doi: 10.1586/1744666X.2014.929944 PMC4117468 24931458

[9] Fekadu J Modlich U Bader P Bakhtiar S. Understanding the role of LFA-1 in leukocyte adhesion deficiency type I (LAD I): moving towards inflammation? Int J Mol Sci. (2022) 23:3578. doi: 10.3390/ijms23073578 35408940 PMC8998723

[10] Hanna S Etzioni A. Leukocyte adhesion deficiencies. Ann N Y Acad Sci. (2012) 1250:50–5. doi: 10.1111/j.1749-6632.2011.06389.x 22276660

[11] Thakur N Sodani R Chandra J Singh V. Leukocyte adhesion defect type 1 presenting with recurrent pyoderma gangrenosum. Indian J Dermatol. (2013) 58:158. doi: 10.4103/0019-5154.108076 PMC3657233 23716823

[12] Bondarenk AV Boyarchuk OR Sakovich IS Polyakova EA Migas AA Kupchinskaya AN. Variable CD18 expression in a 22-year-old female with leukocyte adhesion deficiency type I: clinical case and literature review. Clin Case Rep. (2023) 11:e7791. doi: 10.1002/ccr3.7791 37601427 PMC10432584

[13] Hinze CH Lucky AW Bove KE Marsh RA Bleesing JH Passo MH. Leukocyte adhesion deficiency type 1 presenting with recurrent pyoderma gangrenosum and flaccid scarring. Pediatr Dermatol. (2010) 27:500–3. doi: 10.1111/pde.2010.27.issue-5 20807363

[14] Moutsopoulos NM Zerbe CS Wild T Dutzan N Brenchly L DiPasquale G. Interleukin-12 and interleukin-23 blockade in leukocyte adhesion deficiency type 1. N Engl J Med. (2017) 376:1141–46. doi: 10.1056/NEJMoa1612197 PMC5494261 28328326

[15] Weiss EH Ko CJ Leung TH Micheletti RG Mostaghimi A Ramachandran SM. Neutrophilic dermatoses: a clinical update. Curr Dermatol Rep. (2022) 11:89–102. doi: 10.1007/s13671-022-00355-8 35310367 PMC8924564

[16] McClanahan D Funk T Small A. Sweet syndrome in the pediatric population. Dermatol Clin. (2022) 40:179–90. doi: 10.1016/j.det.2021.12.005 35366971

[17] Heath MS Ortega-Loayza AG. Insights into the pathogenesis of Sweet’s syndrome. Front Immunol. (2019) 10:414. doi: 10.3389/fimmu.2019.00414 30930894 PMC6424218

[18] Marzano AV Borghi A Wallach D Cugno M. A comprehensive review of neutrophilic diseases. Clin Rev Allergy Immunol. (2018) 54:114–30. doi: 10.1007/s12016-017-8621-8 28688013

[19] Cook QS Zdanski CJ Burkhart CN Googe PB Thompson P Wu EY. Idiopathic, refractory Sweet’s syndrome associated with common variable immunodeficiency: a case report and literature review. Curr Allergy Asthma Rep. (2019) 19:32. doi: 10.1007/s11882-019-0864-4 31089823

[20] Elliot SP Mallory SB. Sweet syndrome: an unusual presentation of chronic granulomatous disease in a child. Pediatr Infect Dis J. (1999) 18:568–70. doi: 10.1097/00006454-199906000-00025 10391198

[21] Gray P Bock V Ziegler DS Wargon O. Neonatal sweet syndrome: a potential marker of serious systemic illness. Pediatrics. (2012) 129:e1353–59. doi: 10.1542/peds.2011-1854 22508923

[22] Knipstein JA Ambruso DR. Sweet syndrome in an infant with chronic granulomatous disease. J Pediatr Hematol Oncol. (2012) 34:372–74. doi: 10.1097/MPH.0b013e3182346be1 22246153

[23] Lyon CC Griffiths CEM. Chronic granulomatous disease and acute neutrophilic dermatosis. Clin Exp Dermatol. (1999) 24:368–71. doi: 10.1046/j.1365-2230.1999.00503.x 10564323

[24] O’Regan GM Ho WL Keogan MT Murphy GM. Sweet’s syndrome in association with common variable immunodeficiency. Clin Exp Dermatol. (2008) 34:192–94. doi: 10.1111/j.1365-2230.2008.02814.x 18782323

[25] Sedel D Huguet P Lebbe C Donadieu J Odievre M Labrun P. Sweet syndrome as the presenting manifestation of chronic granulomatous disease in an infant. Pediatr Dermatol. (1994) 11:237–40. doi: 10.1111/j.1525-1470.1994.tb00593.x 7971558

[26] Smith KN Welborn M Monir RL Motaparhti K Schoch JJ. Pediatric pyoderma gangrenosum associated with leukocyte adhesion deficiency type 1: a case report and review of the literature. Pediatr Dermatol. (2022) 40:1086–90. doi: 10.1111/pde.15292 37002583

[27] Bucchia M Barbarot S Reumaux H Piram M Mahe E Mallett X. Age-specific characteristics of neutrophilic dermatoses and neutrophilic diseases in children. J Eur Acad Dermatol Venereol. (2019) 33:2179–87. doi: 10.1111/jdv.15730 31166045

[28] Yamazaki-Nakashimada M Maravillas-Montero JL Berron-Ruiz L Lopez-Ortega O Ramirez-Alejo N Acevedo-Ochoa E. Successful adjunctive immunoglobulin treatment in patients by leukocyte adhesion deficiency type 1 (LAD-1). Immunol Res. (2015) 61:260–68. doi: 10.1007/s12026-014-8619-8 25527966

[29] Hogg N Stewart MP Scarth SL Newton R Shaw JM Law SK. A novel leukocyte adhesion deficiency caused by expressed but nonfunctional beta2 integrins Mac-1 and LFA-1. J Clin Invest. (1999) 103:97–106. doi: 10.1172/JCI3312 9884339 PMC407855

[30] Guan S Tan SM Li Y Torres J Uzel G Xiang L. Characterization of single amino acid substitutions in the β2 integrin subunit of patients with leukocyte adhesion deficiency (LAD)-1. Blood Cell Mol Dis. (2015) 54:177–82. doi: 10.1016/j.bcmd.2014.11.005 25514840

